# Supplementary material for: Re-Evaluation of Chemotherapeutic Potential of Pyoktanin Blue
Source: Medicines (Basel). 2021 Jun 22;8(7):33. doi: 10.3390/medicines8070033 (PMC8305689; doi:10.3390/medicines8070033)
Supplement: Supplementary file 1 [file medicines-08-00033-s001.zip › medicines-1243940-supplementary.pdf]

Supplementary Materials: Re-Evaluation of Chemotherapeutic Potential of Pyoktanin Blue

Hiroshi Sakagami [1,*], Toshiko Furukawa [2], Keitaro Satoh [3], Shigeru Amano [1], Yosuke Iijima [4], Takuro Koshikawa [5], Daisuke Asai [5,6], Kunihiko Fukuchi [7], Hiromu Takemura [5], Taisei Kanamoto [6] and Satoshi Yokose [2]

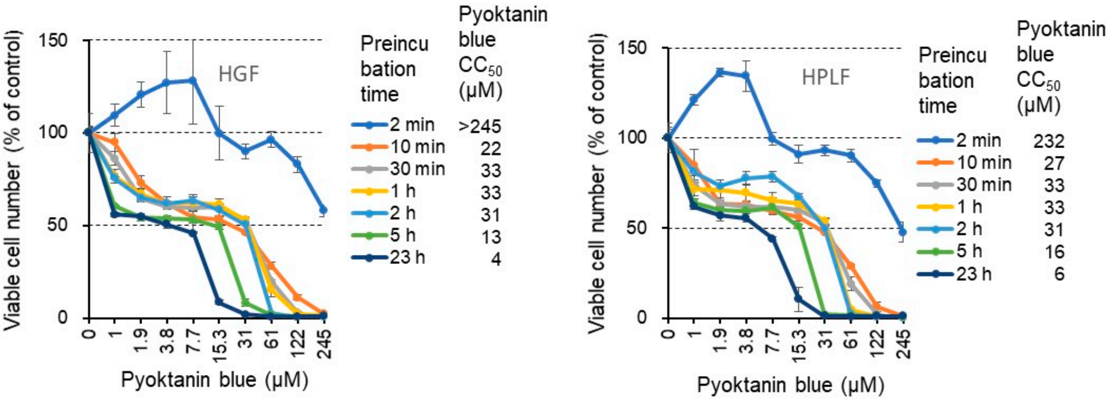

Figure S1. Rapid cytotoxic action of PB. HGF and HPLF cells were pre-incubated for the indicated time in DMEM + 10%FBS, and the incubated in the fresh medium for a total of 48 h. The viable cells were then determined by MTT method. Each value represented as mean ± S.D. of triplicate determination. HGF: Human gingival fibroblast. HPLF: Human periodontal ligament fibroblast.
